# Biometry extraction and probabilistic anatomical atlas of the anterior Visual Pathway using dedicated high-resolution 3-D MRI

**DOI:** 10.1038/s41598-023-50980-x

**Published:** 2024-01-03

**Authors:** Emanuele Pravatà, Andrea Diociasi, Riccardo Navarra, Luca Carmisciano, Maria Pia Sormani, Luca Roccatagliata, Andrea Chincarini, Alessandra Ossola, Andrea Cardia, Alessandro Cianfoni, Alain Kaelin-Lang, Claudio Gobbi, Chiara Zecca

**Affiliations:** 1 Neurocenter of Southern Switzerland, EOC, Neuroradiology, Lugano, Switzerland; 2 Faculty of Biomedical Sciences, Università Della Svizzera Italiana, Lugano, Switzerland; 3 Department of Health Sciences, University of Genova, Genova, Italy; 4 Institute for Advanced Biomedical Technology (I.T.A.B.), Chieti, Italy; 5 Istituto Nazionale Di Fisica Nucleare (INFN), Genova, Italy; 6 Neurocenter of Southern Switzerland, EOC, Ophthalmology, Lugano, Switzerland; 7 Neurocenter of Southern Switzerland, EOC, Neurosurgery, Lugano, Switzerland; 8 Neurocenter of Southern Switzerland, EOC, Neurology, Lugano, Switzerland

**Keywords:** Diagnostic markers, Object vision

## Abstract

Anterior Visual Pathway (aVP) damage may be linked to diverse inflammatory, degenerative and/or vascular conditions. Currently however, a standardized methodological framework for extracting MRI biomarkers of the aVP is not available. We used high-resolution, 3-D MRI data to generate a probabilistic anatomical atlas of the normal aVP and its intraorbital (iOrb), intracanalicular (iCan), intracranial (iCran), optic chiasm (OC), and tract (OT) subdivisions. We acquired 0.6 mm^3^ steady-state free-precession images from 24 healthy participants using a 3 T scanner. aVP masks were obtained by manual segmentation of each aVP subdivision. Mask straightening and normalization with cross-sectional area (CSA) preservation were obtained using scripts developed in-house. A probabilistic atlas (“aVP-24”) was generated by averaging left and right sides of all subjects. Leave-one-out cross-validation with respect to interindividual variability was performed employing the Dice Similarity Index (DSI). Spatially normalized representations of the aVP subdivisions were generated. Overlapping CSA values before and after normalization demonstrate preservation of the aVP cross-section. Volume, length, CSA, and ellipticity index (*ε*) biometrics were extracted. The aVP-24 morphology followed previous descriptions from the gross anatomy. Atlas spatial validation DSI scores of 0.85 in 50% and 0.77 in 95% of participants indicated good generalizability across the subjects. The proposed MRI standardization framework allows for previously unavailable, geometrically unbiased biometric data of the entire aVP and provides the base for future spatial-resolved, group-level investigations.

## Introduction

The normal structure of the anterior Visual Pathway (aVP) may be affected by a variety of acquired conditions, such as glaucoma^1,2^, inflammatory^3,4^, post-traumatic^5^, ischemic^6^, radiation-induced damage^7^, as well as inheritable diseases^8^. Despite several previous efforts^9–13^, there is currently no dedicated methodological framework for the morphometric evaluation of the entire aVP. Optic computed tomography (OCT) allows only for a quantitative assessment of the optic head retinal nerve fiber layer (RNFL), but provides no direct information on the remaining aVP. The very thin and tortuous morphology of the aVP and motion artefacts from eyeball movement pose specific technical challenges for MRI assessment making classical imaging protocols, typically employing two-dimensional (2-D) supra-millimetre slices, suboptimal for a geometrically unbiased assessment.

To address this situation, we developed a dedicated and relatively simple framework for the standardized high-resolution MRI assessment of the entire aVP and its intraorbital (iOrb), intracanalicular (iCan), intracranial (iCran), optic chiasm (OC), and optic tract (OT) anatomical subdivisions. We used three-dimensional (3-D) 0.6 mm^3^ images from a group of 24 healthy participants to develop an image normalization pipeline based on manually segmented data of the normal aVP, in order to: (1) allow for standardized structural analyses, (2) extract salient biometrics, and (3) generate a probabilistic atlas providing statistical knowledge of the aVP morphology.

## Methods

### Study participants

This study was approved by the local ethics committee of Canton Ticino, Bellinzona, Switzerland, (REF: 2017-00814; CE3224). Written informed consent was obtained from all participants before taking part in the study. The study was conducted according to ethical principles laid down by the latest version of the Declaration of Helsinki. Participants were recruited and underwent the MRI acquisition at the Neurocenter of Southern Switzerland between November 2017 and March 2019 as part of a prospective investigation^14^. Inclusion criteria were: (1) healthy male or female, (2) age ≥ 18 and < 65. Exclusion criteria were: (1) history of neurological or (2) ophthalmological diseases (including multiple sclerosis, tumors, glaucoma, retinopathy, maculopathy), (3) spherical refractive errors worse than  − 6D^15^, (4) tumors, (5) severe eye and/or head trauma, (6) pregnancy, (7) claustrophobia. One female subject was excluded from the study due to high myopia (− 8.5D), 24 participants (10 females, 14 males; mean age 35.8 ± 9 years) were included and their scans analyzed.

### Subject preparation and MRI data acquisition

Images were acquired on a single 3 T MRI “Skyra” scanner (Siemens, Erlangen, Germany) using a 64-channel head coil. Several previously proposed procedures were applied to minimize voluntary and/or spontaneous gaze and saccades movements, that have potential to cause image quality degradation^14^. If needed, MRI-compatible eyeglasses were provided to correct for refractive defects. Dimmed room lights optimized visual comfort for the participants. They were asked to lie still during image acquisition and to focus their gaze on a target placed on the scanner gantry in front of their eyes to maintain a straight gaze, or to keep their eyes closed if/when feeling unable to maintain fixation*.*

Imaging data for segmentation consisted of “constructive interference in steady state” (CISS) images covering the entire aVP with the following parameters: TR = 8.1 ms, TE = 3.74 ms, FoV = 150 mm^2^, matrix = 245 × 245, slice thickness = 0.6 mm, acquired voxel size = 0.6 × 0.6 × 0.6 mm^3^, number of slices = 72, scan duration = 3′11″. Images were transferred to a PC workstation running Linux and were checked for major artefacts by an experienced neuroradiologist (E.P.).

#### aVP extraction

**Figure 1:**
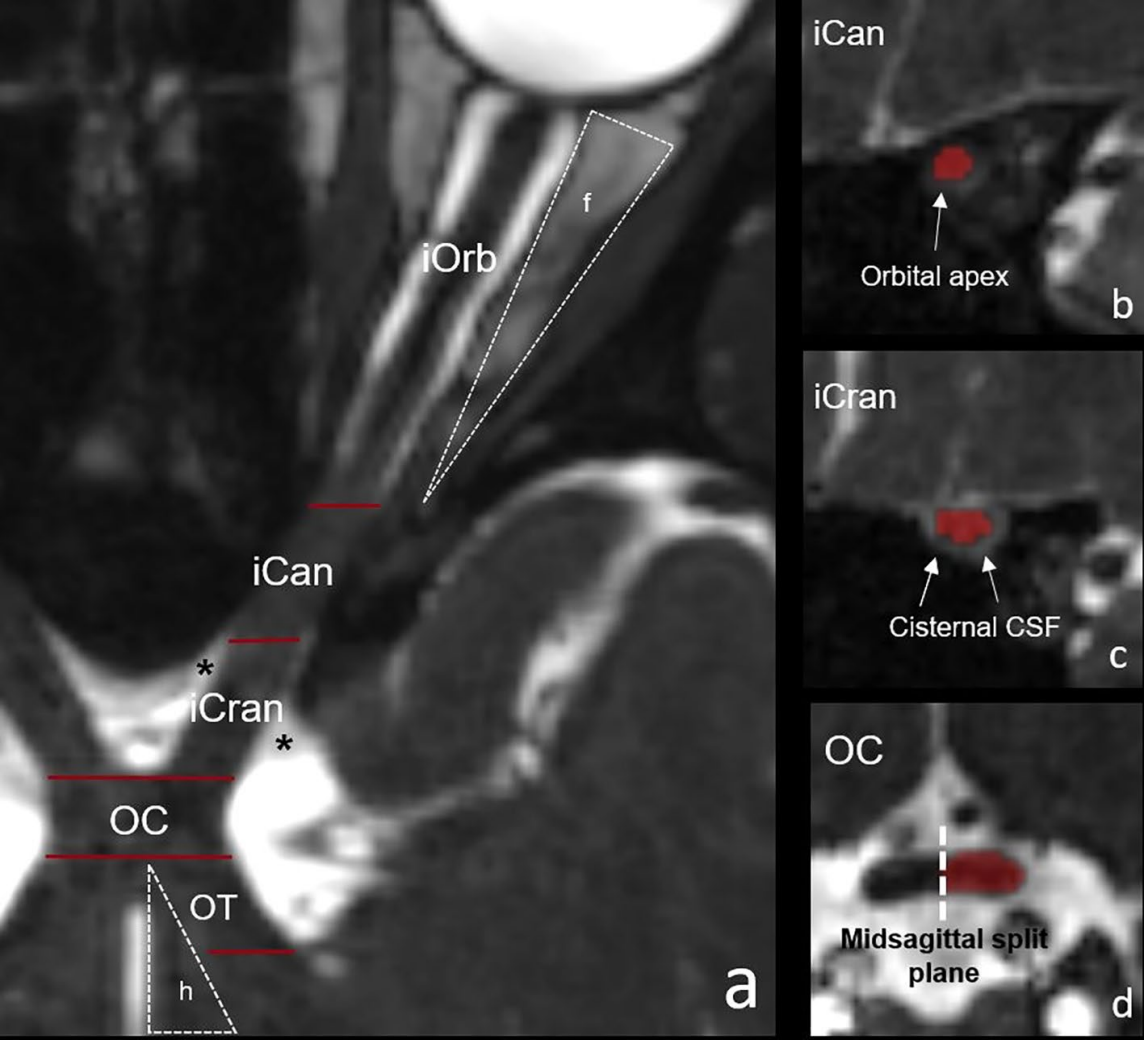
Illustration of the aVP subdivision boundaries applied for segmentation, as seen in one subject on the CISS images. The left panel shows a curved reconstruction of the aVP along its longitudinal axis, with red bars marking the boundaries (**a**). The iOrb/iCan boundary is located at the end of the intraorbital fat (**f**) and corresponds to the orbital apex at the level of the annulus of Zinn envisioned in (**b**). The transition from iCan to iCran is marked by the cisternal CSF (*) surrounding the nerve as seen in (**c**). The OC is the segment where the left and right aVPs merge perpendicular to the midsagittal plane (dashed line in **d**). The hypothalamus (**h**), representing the medial OT boundary, is highlighted in (**a**).

To establish reproducible and MRI-suitable landmarks, we adapted the aVP anatomical subdivision criteria^14^, that were based on several sources of information from previous post-mortem descriptions^16–19^, as illustrated in Fig. 1. Specifically, the subdivisions were:

**iOrb**: from the optic nerve (ON) head to the last section where the orbital fat can be seen surrounding the nerve sheath.

**iCan**: the ON segment coursing within the optic canal, defined as the segment without fat and without cisternal CSF surrounding the nerve.

**iCran**: from the iCan segment end to the anterior OC boundary.

**OC**: the aVP section where the left and right iCran and OT segments merge into a single trunk, and the corresponding sides cannot be separated. For the present study, the OC was split according to the midsagittal plane into the left and right hemi-trunks.

**OT**: from the posterior OC boundary to the lateral geniculate nucleus, defined as the section where no CSF surrounding the nerve can be seen.

Using ITK-SNAP (v.3.6.0 freely available at www.itksnap.org), a neuroradiologist in training (A.D.) labelled each aVP subdivision on the CISS images according to the anatomical landmarks described above under the constant supervision of an experienced neuroradiologist (E.P.). Readers had the post-mortem normal anatomy descriptions constantly on hand for reference. Accuracy was optimized by iteratively revising segmentation until agreement with the senior neuroradiologist was achieved. The labels were saved as separate binary masks for each subdivision and side.

#### Image normalization pipeline

**Figure 2:**
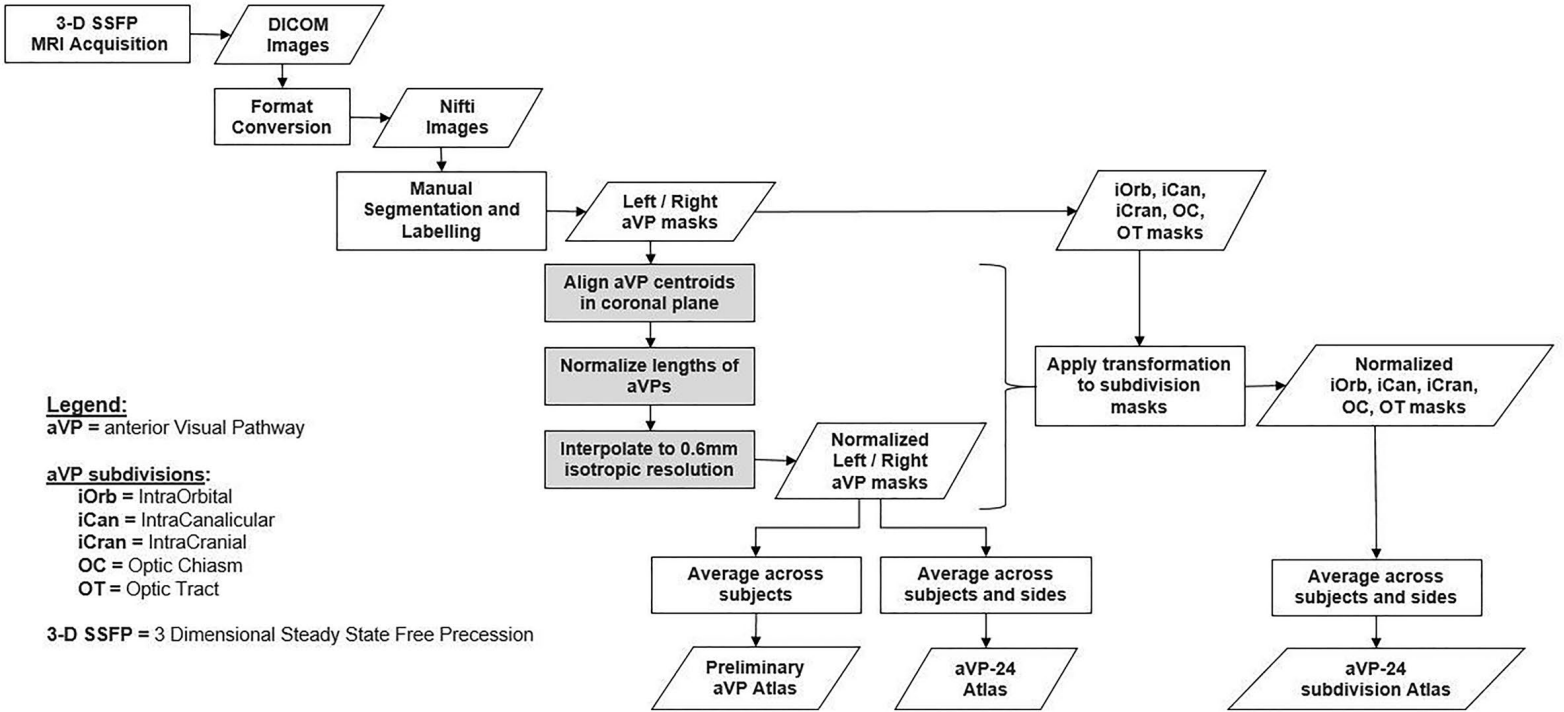
Flow-chart of consecutive steps involved in image normalization and creation of the aVP-24 atlas.

**Figure 3:**
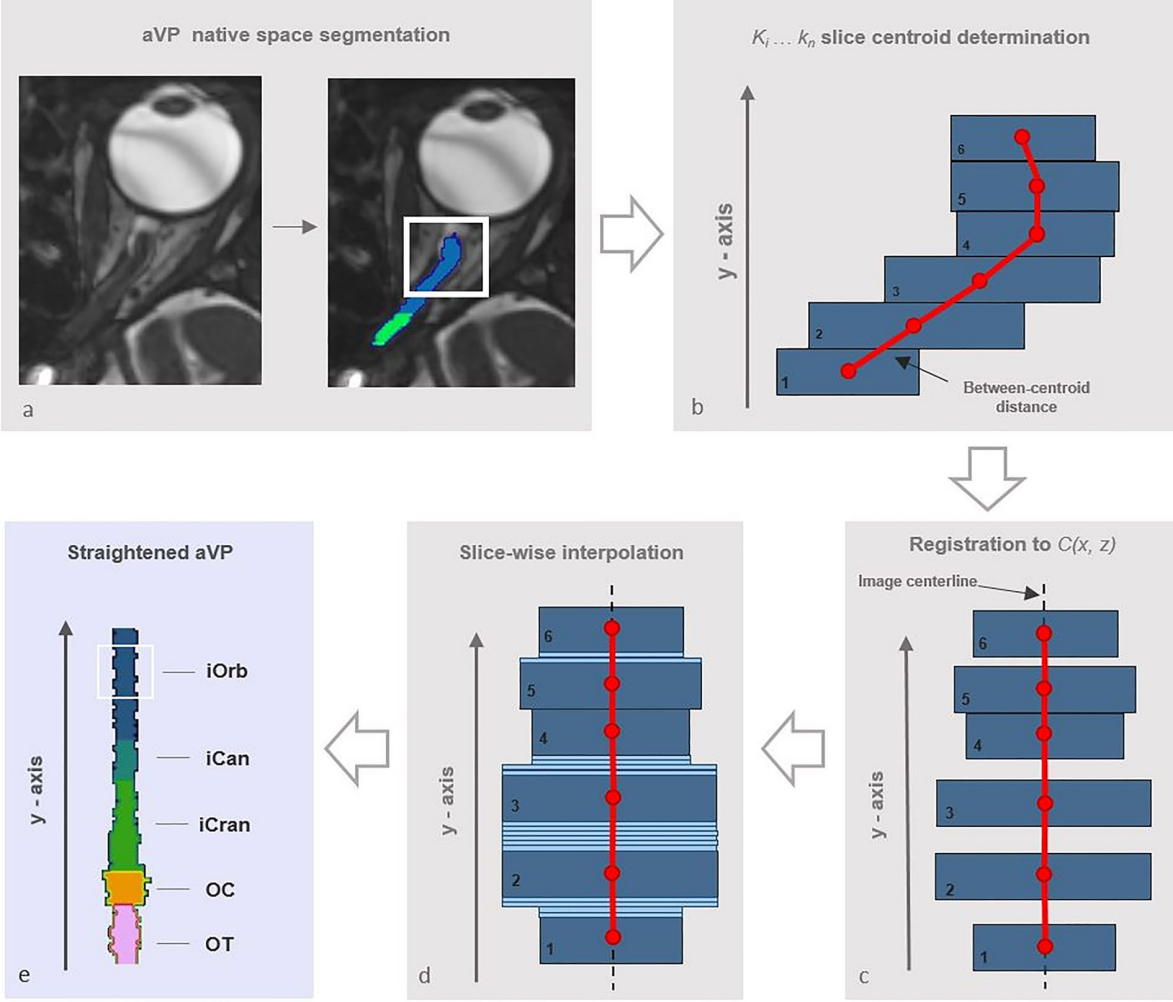
Diagram illustrating the aVP straightening process. Following manual segmentation in the native space (**a**), the slice centroids and the corresponding between-centroid distance were calculated (red markers in **b**). Then, slices were linearly shifted on the (x, z) transverse plane to the image centerline (red line in **c**), with preservation of the original between-centroid distance and CSA. The resulting gaps between shifted slices were filled by slice-wise interpolation, preserving the average CSA (light-blue slices in **d**). Finally, the straightened iOrb, iCan, iCran, OC and OT sections were concatenated along the image centerline (**e**). The (**b**–**d**) sample sketches are simplified for illustrative purposes.

The image normalization pipeline steps are indicated in gray in Fig. 2. Its first step consisted of aligning the centroids of the label masks in the coronal plane, with the effect of straightening the ON (Fig. 3). Each aVP segment mask and side were processed separately in an identical manner. For each *k*_*i*_ labelled slice orthogonal to the y axis (representing the image anterior–posterior axis orthogonal to the coronal plane), the centroids and the corresponding D_i, i+1_ distance between them in the k_i_, k_i+1_ slices were calculated. Then, the segments length (TL) was calculated as:

1 $${TL=\sum }_{i=1}^{n}{D}_{i}$$

Each k_i_ slice was linearly shifted to the image C(x, z) center, so that:

2 $$\left(\Delta x,\Delta z\right)=\left(\frac{Nx}{2}-Cx,\frac{Nz}{2}-Cz\right)$$

where N_i_ corresponds to the mask centroid and C_i_ the image center for the j = x-z-axis. Following shifting, if $\left(|\Delta x|,|\Delta z|\right)>0$, the straightened distance between consecutive centroids is smaller than D_i, i+1_. To preserve aVP length, we performed slice-wise interpolation, by ten-fold replication (in effect creating a 10^−1^ (y) coronal slice thickness (i.e. 0.06 mm)) slice, and introducing sufficient slices by nearest neighbor replication to counter the reduction of straightened distance. This procedure was repeated for all slices, ensuring the same TL for the straightened aVP subdivisions.

After assembling all the aVP subdivisions (iOrb, iCan, iCran, OC, OT) of the same side, linear normalization with respect to the y-axis was performed to rescale each aVP length to the average TL (61.1 mm) across all subjects and sides. For each subject and aVP side, the total length (TL_i_) was temporarily increased to a nominal length by linearly increasing the D_i, i+1_ and maintaining proportionality of each subdivision, applying the same interpolation procedure as for straightening (see above). Importantly, the CSA of each slice generated from an original slice, was preserved. After the TL was adapted, the final voxel size resampled to an isotropic 0.6 mm^3^ resolution.

All procedures were conducted on the manually segmented label masks using scripts developed in-house with Matlab (v.R2016a) and its Image Processing toolbox (MathWorks, Natick, USA, https://www.mathworks.com), as well as fslutils (v6.0.5, Analysis Group, FMRIB, Oxford, UK, https://fsl.fmrib.ox.ac.uk), to allow for a slice-wise comparison of the cross-sectional area (CSA) between subjects.

#### Probabilistic atlas creation

The probabilistic atlas was created by taking the voxel-wise average of all the binary normalized aVP masks, i.e. all the L and R-to-L flipped masks, for a total of N = 48 aVP masks and multiplying by 100 to obtain percentage values in the range of 0–100. Therefore, each resulting voxel intensity represented the overlap across the 48 aVP of the 24 participants in terms of probability of the atlas voxels to belong to all participants’ aVP. The image origin was set at the centerline of the most anterior iOrb section corresponding to the nerve head. Individual atlases for each corresponding segment of the iOrb, iCan, iCran, OC, and OT subdivisions were also generated applying the same procedure (Fig. 2). To estimate the impact of interindividual anatomical variability on the atlas morphology, and thus to estimate the atlas generalizability, we used a leave-one-out approach by testing the similarity between each subject’s normalized aVP segmentation and the atlas with all subjects’ aVP averaged except for that particular one to be tested. Similarity was estimated with the Dice Similarity Index (DSI) defined as

3 $$DSI\left(A, B\right)=2 \frac{{\text{A}}\cap {\text{B}}}{{\text{A}}+{\text{B}}}$$

where A represents the individual subject aVP, B the atlas’s aVP with threshold at 50% probability, and ∩ their intersection.

#### aVP biometry

Four biometric variables were extracted: volume, length, mean cross-sectional area, and ellipticity index (*ε*), separately for the left and right side. (1) Volume was directly obtained from the CISS images label masks (see the “aVP extraction” section above). (2) Length was obtained by estimating the TL distance between all aVP slice centroid, (see “Image Normalization pipeline” section above). (3) mCSA was calculated adapting a method described for the spinal cord^20,21^ by dividing each segment volume by its length. (4) *ε* estimates the degree to which the nerve section approaches an elliptical configuration applying the formula:

4 $$\varepsilon =\frac{{h}_{1}-{h}_{2}}{{h}_{1}}$$

where h1 > h2 represents the two orthogonal cross-sectional hemi-axes, as reported from post-mortem studies^17,22,23^. Values close to 0 indicate a circular shape, whereas values close to 1 an elliptical configuration.

### Statistical analysis

Descriptive statistics (mean with standard deviation, minimum and maximum, and 95% confidence interval for the variables with Gaussian distribution; median with the first and third quartile for the corresponding variables with non-Gaussian distribution) were calculated for continuous variables. For categorical variables, counts and percentages were provided. Left side vs Right side measurements were compared with paired t-tests. Linear Mixed-Effects Models were applied to investigate associations between the measurements (dependent variable) and the demographic characteristics (i.e., age and sex) while accounting for intra-subject variability. Mixed models allowed estimation of the between-subject proportion of the measurement variance. The coefficient of variation, describing the normalized between-subject standard deviation relative to the mean, was used to compare inter-subject segment variability. Between-subject variability was assessed by defining variables obtained from the absolute raw measurements by standardizing the volume and the length of each segment on the total aVP values (i.e., the proportion of aVP located in the segment), while mCSA and *ε* were standardized on the corresponding mean aVP values (i.e., the variation of each segment from a hypothetical nerve with same volume, the same length but a constant mCSA/*ε*). *P*-values < 0.05 of two-tailed tests were considered statistically significant.

## Results

### Standardization pipeline

**Figure 4:**
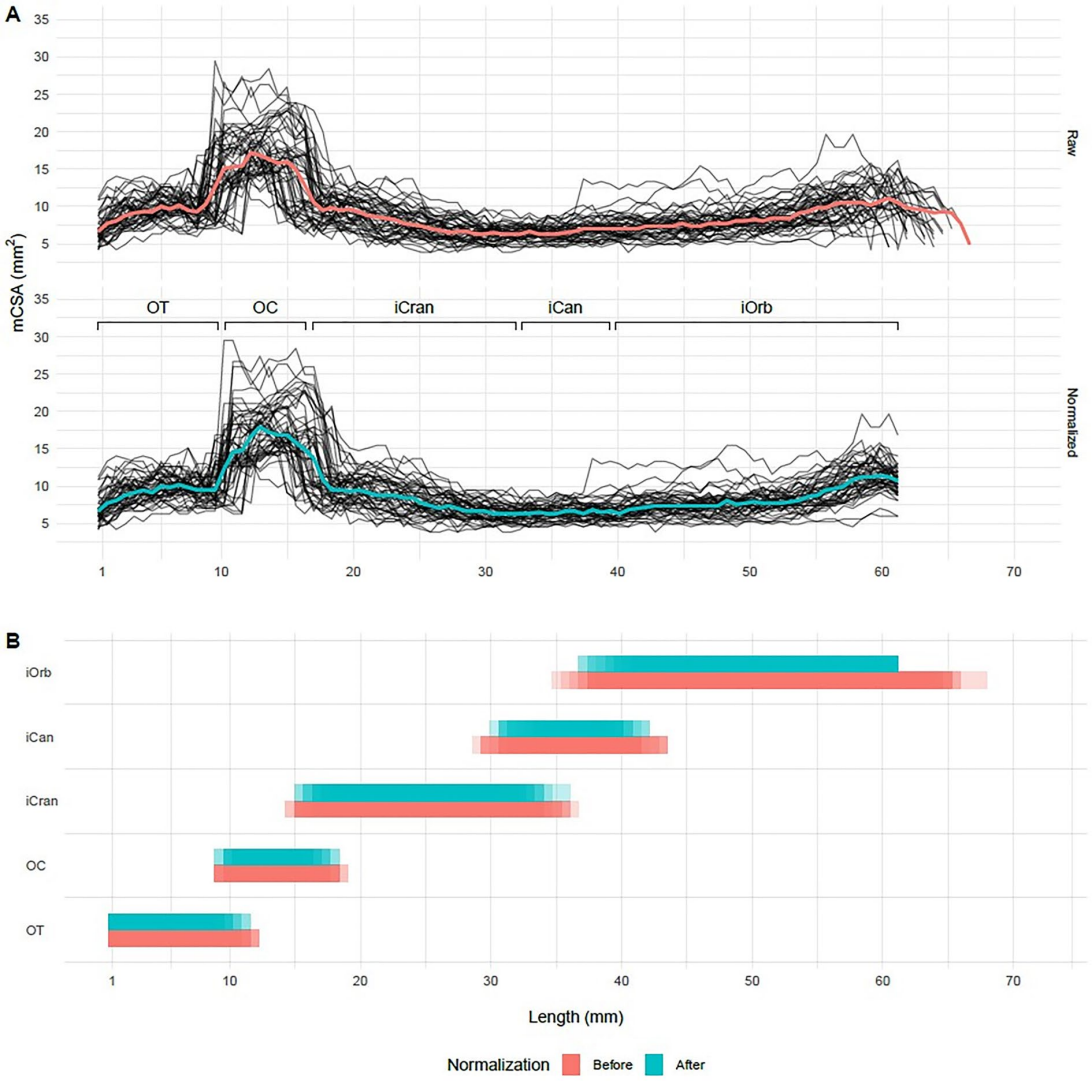
Results of the normalization procedure. (**A**) The CSA for each subject before (upper) and after normalization (lower), as a function of the distance (mm) from the brain showed different length values between subjects, due to the expected anatomical interindividual variability. The corresponding median values are presented in red and cyan colors and demonstrate a good agreement indicating CSA preservation. (**B**) The deformations due to the normalization pipeline, as a function of position/subdivision along the y-axis for each section, comparing the length before (red) and after (cyan) normalization of each subject’s values. The number of individual lengths are shown as bands with fixed transparency levels; areas with higher transparency represent fewer number of subjects.

For each subject, aVP was deformed in the y-axis to match the same length corresponding to the average of group and side (i.e. 61.1 mm). The “spaghetti” diagrams in Fig. 4 show how normalization was applied to the y-axis without affecting the CSA throughout the nerve (Fig. 4A), while preserving each subdivision’s proportional length (Fig. 4B).

### aVP biometry

Table 1 displays the aVP volume, length, mCSA and *ε* biometric data. Concurring with pathology studies^17^, there was no statistically significant difference between the left and right side for each parameter (P range 0.249–0.931). Therefore, side was not included in the subsequent analyses, and all subsequent metrics represent averaged left, right values. The mCSA was larger in the iOrb (7.67 mm^2^) and iCran (6.92 mm^2^) than in the iCan (5.62 mm^2^) subdivision (*P* < 0.001 for both). The OC, split into left and right hemi-trunks, presented the largest mCSA (14.37 mm^2^) subdivision, being almost twice as big than the contiguous iCran (6.92 mm^2^ *P* = 0.018) subdivision. *ε* was significantly different across all segments: (*P* < 0.001). The lowest index was found in iOrb (0.15) indicating an almost circular cross-section, and the highest in OC (0.57), corresponding to an elliptical cross-section.

**Table 1:** aVP biometric data (aVP volume, length, mCSA and *ε* morphometrics) summarized by segment and side.

Metric	Segment	Left	Right	Mean left–right difference*
aVP volume [mm^3^], mean (SD)	ON	319.02 (50.76)	319.14 (50.96)	− 0.12 (− 2.15, 1.91)
	iOrb	172.48 (36.58)	172.61 (37.15)	− 0.14 (− 1.94, 1.66)
	iCan	38.82 (7.02)	39.07 (6.59)	− 0.25 (− 0.90, 0.40)
	iCran	107.73 (16.77)	107.46 (16.75)	0.27 (− 0.30, 0.83)
	OC	94.18 (21.36)	94.17 (21.73)	0.01 (− 1.63, 1.65)
	OT	72.55 (12.04)	72.33 (12.26)	0.22 (− 0.12, 0.57)
	Entire aVP	485.76 (75.41)	485.65 (74.89)	0.11 (− 2.53, 2.76)
Length [mm], mean (SD)	ON	45.27 (2.28)	45.03 (4.51)	0.24 (− 1.15, 1.64)
	iOrb	22.40 (2.18)	22.38 (2.64)	0.01 (− 0.67, 0.70)
	iCan	6.87 (0.66)	7.19 (2.06)	− 0.33 (− 1.10, 0.44)
	iCran	16.01 (1.85)	15.45 (1.80)	0.56 (0.26, 0.86)
	OC	6.53 (0.82)	6.53 (0.82)	0.00 (− 0.16, 0.17)
	OT	9.57 (1.06)	9.46 (0.97)	0.11 (− 0.07, 0.29)
	Entire aVP	61.37 (2.73)	61.02 (4.90)	0.36 (− 0.97, 1.68)
mCSA [mm^2^], mean (SD)	ON	7.04 (1.04)	7.11 (1.13)	− 0.07 (− 0.24, 0.10)
	iOrb	7.65 (1.14)	7.70 (1.34)	− 0.05 (− 0.27, 0.17)
	iCan	5.66 (0.81)	5.59 (1.02)	0.06 (− 0.21, 0.33)
	iCran	6.82 (1.35)	7.03 (1.29)	− 0.21 (− 0.33, − 0.08)
	OC	14.33 (2.10)	14.42 (2.71)	− 0.09 (− 0.57, 0.39)
	OT	7.58 (0.91)	7.64 (0.98)	− 0.06 (− 0.19, 0.07)
	Entire aVP	8.41 (1.06)	8.48 (1.16)	− 0.07 (− 0.19, 0.05)
*ε*	ON	0.26 (0.04)	0.27 (0.04)	− 0.01 (− 0.02, 0.01)
	iOrb	0.15 (0.03)	0.15 (0.04)	− 0.00 (− 0.02, 0.01)
	iCan	0.20 (0.08)	0.21 (0.07)	− 0.02 (− 0.05, 0.02)
	iCran	0.43 (0.06)	0.43 (0.07)	0.00 (− 0.02, 0.03)
	OC	0.57 (0.08)	0.57 (0.08)	0.00 (− 0.02, 0.02)
	OT	0.40 (0.07)	0.38 (0.07)	0.01 (− 0.01, 0.03)
	Entire aVP	0.35 (0.04)	0.35 (0.04)	− 0.001 (− 0.01, 0.01)

*aVP* anterior visual pathway; *ε* ellipticity index; *iCan* intracanalicular segment; *iCran* intracranial segment; *iOrb* intraorbital segment; *mCSA* mean cross section area; *OC* optic chiasm; *ON* optic nerve; *OT* optic tract; *SD* standard deviation.

*Positive values indicate Left > Right, and vice-versa.

Between-subject variability estimates for aVP volume, length, mCSA, and *ε* are reported in Table 2. The relative mCSA had the lowest between-subject coefficient of variation (CV) (average CV per segment 9.5%, range 6.9– − 10.7%), suggesting that this metric was the least influenced by between-subject anatomical differences in our population. Relatively higher CV values were obtained for a VP volume (average CV per sector 11.1%, range 9.9–12.8%), length (average CV per segment 10.0%, range 7.1–12.3%) and *ε* (average CV per segment 13.7%, range 7.9–24.1%).

**Table 2:** aVP biometric interindividual variability estimates for aVP volume, length, mCSA and *ε* morphometrics.

Metric	Segment	Proportion of aVP % (95%CI)	CV^†^ (%)
aVP volume [mm^3^]*	iOrb	35.4 (33.8, 37.0)	11.0
	iCan	8.0 (7.7, 8.4)	9.9
	iCran	22.3 (21.3, 23.2)	10.3
	OC	19.3 (18.3, 20.3)	12.8
	OT	15.0 (14.3, 15.7)	11.3
Total volume ON [mm^3^]		65.7 (64.7, 66.7)	3.8
Length [mm]*	iOrb	36.6 (35.5, 37.6)	7.1
	iCan	11.4 (10.9, 12.0)	12.3
	iCran	25.7 (24.7, 26.8)	10.0
	OC	10.7 (10.2, 11.2)	11.3
	OT	15.6 (15.0, 16.1)	9.2
Total ON length [mm]		73.7 (73.1, 74.4)	2.2
		Variation from mean aVP % (95%CI)	
mCSA [mm^2^]	iOrb	1.4 (− 1.4, 4.2)	6.9
	iCan	− 25.6 (− 28.2, − 22.9)	8.9
	iCran	− 8.6 (− 12.6, − 4.6)	10.9
	OC	90.4 (82.5, 98.3)	10.3
	OT	1.3 (− 3.0, 5.7)	10.7
Overall ON mCSA [mm^2^]		− 6.6 (− 7.3, − 5.9)	1.9
*ε*	iOrb	− 56.6 (− 59.5, − 53.8)	16.6
	iCan	− 41.9 (− 47.5, − 36.3)	24.1
	iCran	23.4 (19.3, 27.6)	8.4
	OC	63.0 (57.9, 68.1)	7.9
	OT	12.0 (6.9, 17.2)	11.5
Overall ON *ε*		− 35.4 (− 38.2, − 32.7)	10.7

*aVP* anterior visual pathway; *ε* ellipticity index; *iCan* intracanalicular segment; *iCran* intracranial segment; *iOrb* intraorbital segment; *mCSA* mean cross section area; *OC* optic chiasm; *ON* optic nerve; *OT* optic tract; *SD* standard deviation.

*Volume and length estimates are relative to the total aVP while mCSA and Ellipticity to the mean aVP.

^†^Coefficient of variation represents the proportion of the estimate variability in relation to its mean (using the standard deviation of the fixed effect terms of the segment estimation model).

### aVP-24 probabilistic atlas

**Figure 5:**
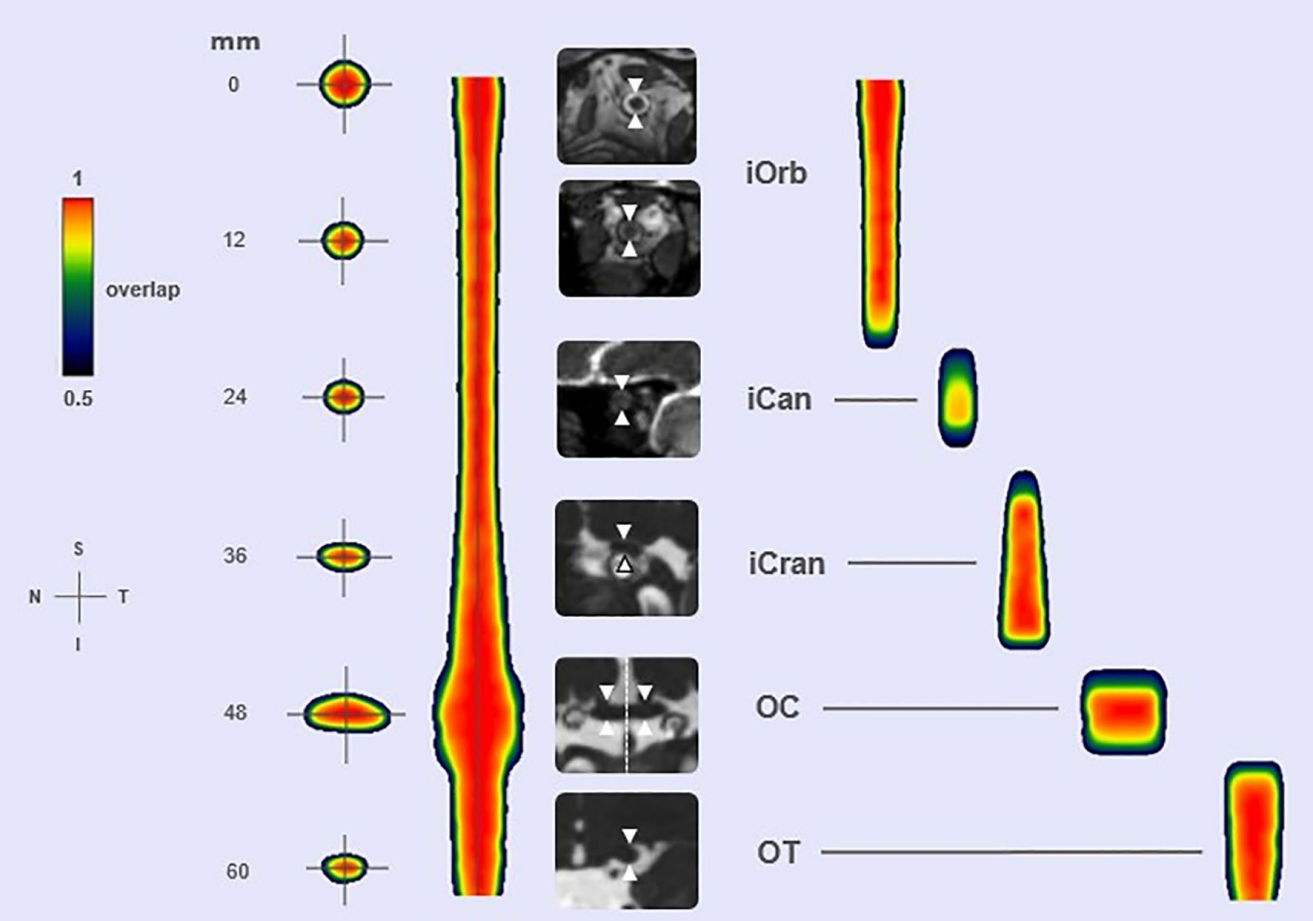
The aVP-24 probabilistic atlas with its iOrb, iCan, iCran, OC and OT anatomical subdivisions seen as coronal (leftmost) and long-axis sections. The millimetre scale indicates the distance from the optic nerve head (which was set as the atlas origin), the color scale the spatial overlap between the 48 nerves: cooler colors indicate lower overlap while warmer colors greater overlap. White arrows highlight the aVP on illustrative coronal oblique CISS images for each corresponding subdivision of the aVP from one participant. The dashed line on the OC coronal image indicates the midline plane employed for splitting. Orientation marker abbreviations: I = inferior, N = nasal, S = superior, T = temporal.

The results of the normalization procedure and probabilistic atlas generation for the aVP and its subdivisions constitute the *anterior Visual Pathway-24* (aVP-24) atlas and are depicted in Fig. 5 in color scale. Each color represents the probability that the voxel belongs to the aVP of all participants. The leave-one-out cross-validation yielded DSI scores of 0.85 in 50% and 0.77 in 95% of the participants (Supplementary Figure S1) and thus shows a good capacity to represent the aVP general anatomy, as the scores obtained correspond to good levels of similarity^24^. At the same time, the 0.77 score provides an estimate of the maximum allowed spatial level that two different populations may share, to permit still the detection of differences when compared.

Consistent with the metrics reported for the anatomical subdivisions in Table 1, the atlas further illustrates the smooth decrease of CSA from the retrobulbar iOrb to the iCan section and a slight increase in the iCran section, to raise sharply at the iCran-OC transition and to fall sharply at the OC-OT transition. The overall shape as estimated by *ε* is almost circular in the extra-cranial segments but becomes increasingly elliptical in the intracranial segments, mostly in the OC.

Following OC split and centerline registration, the OC is represented collinearly with respect to the adjacent iCran and OT segments.

The atlas also provides information about the probability of anatomical subdivision boundaries, highlighting the underlying interindividual differences. The color-coded boundary distribution spread (Fig. 5) illustrates that the iCan segment exhibits the largest boundary uncertainty, being consistent with the known interindividual optic canal length variability^17^.

## Discussion

We developed a dedicated framework for high-resolution and geometrically unbiased evaluation of images of the entire aVP, acquired by MRI. Three main results emerged. First, a relatively simple pipeline including aVP straightening and normalization allows MRI data preparation for standardized group-level analyses. Second, the salient biometry characteristics of the normal aVP and its subdivisions could be extracted. Third, the aVP-24 atlas was developed to provide researchers with probabilistic knowledge of the general aVP MRI morphology of healthy people.

This is a preparatory work to be later expanded to quantitative, standardized investigations about aVP pathological conditions, for example the degree of modification damage related to different optic neuropathies such as CSA degree and its spatial distribution.

The peculiar aVP anatomy, characterized by sharp bends and varying perioptic tissues along its course, hinders any straightforward application of the methodologies currently employed for the brain and/or spinal cord. To the best of our knowledge, the present pipeline is the first one especially dedicated to the assessment of the entire aVP morphometry. One strength is the employment of a specific MRI acquisition technique that yields optimal nerve-to-CSF contrast, high-resolution, and geometrically unbiased tissue sampling in a reasonable scan time (about three minutes). The employed CISS images are based on the “steady-state free-precession” technique, which provides optimal signal contrast at the interface between the cranial nerves and the surrounding cerebrospinal fluid (CSF)^25^. Another strength is the relatively simple normalization pipeline preserving the cross-section aVP topology, thus, allowing spatially standardized comparisons between groups.

Previous morphometric MRI studies were mostly limited to the ON and/or provided 1-D or 2-D metrics, and/or employed non-dedicated imaging protocols (Table 3). In two studies including healthy subjects, dedicated ultrafast 2D MRI “HASTE” images with 3 mm and 2 mm slice thickness were employed to obtain 1-D and CSA measurements of the iOrb at different arbitrary points^9,12^. In a small group of 8 healthy subjects a semiquantitative segmentation of 2D T2-TSE images were acquired using a multidynamic scheme to obtain measurements limited to 8–10 slices of the intraorbital ON^13^. In another clinical study comparing 23 healthy subjects with a group of patients with idiopathic intracranial hypertension, volume calculations of the intraorbital ON was based on 2D T2-weighted 2 mm-thick images^11^. Finally, in a clinical study including a small group of 12 healthy subjects and 8 patients with glaucoma, an automated volumetry analysis of the aVP using standard 3-D-MPRAGE images and a non-dedicated processing pipeline was performed^10^. Contrarily to the previous MRI reports and pathology studies^17^, however, a substantial volume difference between the L and R ON and OT in healthy subjects was reported, which is suggestive for underlying biases related to the non-dedicated pipeline.

**Table 3:** Summary of the biometry reports available from previous MRI studies.

Reference	Healthy subjects (N)	Field Strength	Slice thickness (mm)	Metric dimensions	Intraorbital ON cross sectional diameter (mm)	Intraorbital ON cross sectional area (mm^2^)	Intraorbital ON volume (mm^3^)	ON volume (mm^3^)	OC volume (mm^3^)	OT volume (mm^3^)
Bäuerle et al.^29^	15	3 T	3	1-D	5.69 ± 0.77*	–	–	–	–	–
Kim et al.^30^	314	1.5 T	(TOF-MRA)	1-D	4.71 ± .31	–	–	–	–	–
Furlanetto et al.^9^	24	3 T	2	1-D	3.29 ± 0.4^†^; 8.51 ± 1.6^†^	–	–	–	–	–
Geeraerts et al.^31^	36	3 T	4	1-D	5.08 ± 0.71*	–	–	–	–	–
Karim et al.^27^	46	1.5 T	2	1-D	3.50 ± 0.4^§^	–	–	–	–	–
Kashiwagi et al.^32^	23	1.5 T	3	1-D	2.47 ± 0.24	–	–	–	–	–
Lagreze et al.^12^	33	3 T	3	1-D	3.23*; 2.94^‡^; 2.67 mm^§^	–	–	–	–	–
Liu et al.^33^	413	3 T	(TOF-MRA)	3-D	4.76 ± 0.43*	–	–	–	–	–
Votruba et al.^34^	10	1.5 T	3	1-D	3.5 ± 0.3 (ant part); 3.1 ± 0.3 (middle part); 3.1 ± 0.3 (post part)	–	–	–	–	–
Seitz et al.^35^	9	N/A	2	1-D	5.2 ± 1.11*	–	–	–	–	–
Weigel et al.^36^	32	3 T	5	1-D	3.23 ± 0.23 (ant part); 2.9 ± 0.3 (middle part); 2.7 ± 0.3 (post part)	–	–	–	–	–
Zhank et al.^37^	30	3 T	4	1-D	2.55 ± 0.37^†^; 2.32 ± 0.32^‡^; 2.2 ± 0.25^§^	–	–	–	–	–
Yiannakas et al.^38^	18	1.5 T	3	2-D	–	8.5 ± 1.7*	–	–	–	–
Yiannakas et al.^13^	16	3 T	N/A	2-D	–	5.0 ± 0.7* (left); 5.3 ± 0.8* (right)	–	–	–	–
Hernowo et al.^10^	12	3 T	1 mm (3-D)	3-D (automated)	–	–	137.6 ± 17.6 (left); 94.6 ± 17.1 (right)	–	42.8 ± 12.15 (L + R average)	417.4 ± 41.5 (right); 561.5 + / − 51.5 (left)
Hoffman et al.^11^	23	1.5 T	2	3-D	–	–	194.03 ± 47.77 mm^3^	–	–	–
Ramli et al.^39^	30	3 T	1.2	3-D	–	–	–	297.80 ± 71.45 (left); 296.56 ± 71.02 (right)	–	–

Distance from eye bulb: * = 3 mm; † = 5 mm; ‡ = 10 mm; § = 15 mm.

We can provide a systematic description of the salient morphometry features of the entire aVP for the first time. These data are in line with the descriptions available from pathology studies^17,26,27^. In particular, the average ON length estimated in our study is quite close to that reported by Radunovic et al.^17^ and the iOrb mCSA diameter is consistent with reported average cross-section diameters^26^. However, whereas the iOrb mCSA (L/R average = 7.67 mm^2^) diameter approaches the surface area measured at the level of the orbital foramen (L/R average = 6.97 mm^2^)^17^, our estimate of the iCan was substantially smaller (L/R average = 5.62 mm^2^) when compared to the cranial foramen measurements in cadavers (8.76 mm^2^)^17^. We speculate that such discrepancies might be related, at least in part, to (1) limitations in the iCan contouring accuracy because of the reduced signal contrast gradient related to the perioptic CSF scarcity at this level and (2) methodological differences in the determination of the iCan boundaries. Supporting such hypotheses, our iCan MRI volume estimates (L/R average = 38.95 mm^3^) are substantially smaller compared to the pathology data (50.25 mm^3^)^17^.

The description of the aVP cross-section ellipticity is another novelty of the present study is. *ε* increases from an almost circular to an elliptical configuration from the intraorbital to the intracranial subdivisions. This is in line with post-mortem reports^17,22^, and parallels the configuration of the osseous optic canal^17^*.* Another interesting finding was that the OC exhibited the largest mCSA (L/R average = 14.37 mm^2^) and the highest *ε* (L/R average = 0.57). This is consistent with the general appearance of the OC on standard MRI as well as with reports from gross anatomy^17^, and is reasonably linked to the arrangement of the fibers hemidecussation^28^.

The aVP-24 atlas provides probabilistic knowledge of the general aVP morphology and its anatomical subdivisions and might allow localization of differences in the morphology, trophism and/or lesion distribution between healthy and/or diseased populations in a future extension of this study. Spatial localization of the differences may be obtained (1) in terms of distance in mm from the atlas origin, which was set at the optic nerve head, or (2) in terms of categorization within the different aVP subdivisions. The atlas additionally discloses the aVP general morphology visible in vivo on MRI being characterized by CSA and ellipticity variation, consistent with post-mortem findings^17,27^. Remarkably, boundary uncertainty was highest for the iCan segment, corresponding to a low spatial localization reliability when studying a group. Two assumptions underlying the aVP-24 atlas creation must be addressed. First, the complex OC geometry was assimilated to that of the remaining aVP by splitting the OC into hemi-trunks. This allowed for methodology harmonization and simplification but led to the artefactual “banjo-like” shape related to the shift of the relatively larger OC slices to the centerline (Fig. 5). The other assumption concerning the left- and right-side symmetry was based on the lack of significant differences in the biometry data detected in our population. These assumptions led to the proposed unpaired atlas generated by straightening and averaging the L and right aVP sides together.

Several limitations need to be acknowledged. First, segmentation was performed manually being time consuming and limiting potential reproducibility. Future development of automated segmentation would be a desirable direction of development. Second, our data was obtained from a relatively small number of healthy adult subjects. Larger studies are warranted to provide age and sex specific normative data. Third, *ε* might have been overestimated particularly in sharp-angled segments, because a centerline slice shift was conducted according to the image coronal plane upon nerve straightening. Finally, the unpaired atlas configuration implies the loss of the visual field topology information, limiting correlations with functional deficits.

### Supplementary Information

Supplementary Information.

## Data Availability

The aVP-24 atlas and the original software developed in this study are made freely available online as part of the 'aVP-Toolbox' project at https://github.com/EmanuelePravata/aVP-Toolbox.

## References

[1] Quigley HA, Miller NR, George T (1980). Clinical evaluation of nerve fiber layer atrophy as an indicator of glaucomatous optic nerve damage. Arch. Ophthalmol..

[2] Smith CA, Vianna JR, Chauhan BC (2017). Assessing retinal ganglion cell damage. Eye.

[3] Dutra BG, da Rocha AJ, Nunes RH, Maia ACMJ (2018). Neuromyelitis optica spectrum disorders: Spectrum of MR imaging findings and their differential diagnosis. Radiographics.

[4] Harrigan RL (2017). Quantitative characterization of optic nerve atrophy in patients with multiple sclerosis. Mult. Scler. J.- Exp., Transl. Clin..

[5] Sarkies N (2004). Traumatic optic neuropathy. Eye.

[6] Biousse V, Newman NJ (2015). Ischemic optic neuropathies. N. Engl. J. Med..

[7] Danesh-Meyer HV (2008). Radiation-induced optic neuropathy. J. Clin. Neurosci..

[8] Newman NJ, Biousse V (2004). Hereditary optic neuropathies. Eye.

[9] Furlanetto RL (2018). Structural and functional analyses of the optic nerve and lateral geniculate nucleus in glaucoma. PloS one.

[10] Hernowo AT, Boucard CC, Jansonius NM, Hooymans JM, Cornelissen FW (2011). Automated morphometry of the visual pathway in primary open-angle glaucoma. Invest. Ophthalmol. Vis. Sci..

[11] Hoffmann J (2014). Volumetric assessment of optic nerve sheath and hypophysis in idiopathic intracranial hypertension. AJNR Am. J. Neuroradiol..

[12] Lagrèze WA (2007). Morphometry of the retrobulbar human optic nerve: comparison between conventional sonography and ultrafast magnetic resonance sequences. Invest. Ophthalmol. Vis. Sci..

[13] Yiannakas MC (2013). MRI acquisition and analysis protocol for in vivo intraorbital optic nerve segmentation at 3T. Invest. Ophthalmol. Vis. Sci..

[14] Pravatà E (2021). Dedicated 3D-T2-STIR-ZOOMit imaging improves demyelinating lesion detection in the anterior visual pathways of patients with multiple sclerosis. AJNR Am. J. Neuroradiol..

[15] Flitcroft DI (2019). IMI-defining and classifying myopia: A proposed set of standards for clinical and epidemiologic studies. Invest. Ophthalmol. Vis. Sci..

[16] Rabrenović M (2015). Neurotoxic effects of oxygen in hyperbaric environment: A case report. Vojnosanit. Pregl..

[17] Radunovic M (2019). Morphometric characteristics of the optic canal and the optic nerve. Folia Morphol..

[18] Saeki N (2004). Histologic characteristics of normal perivascular spaces along the optic tract: New pathogenetic mechanism for edema in tumors in the pituitary region. AJNR Am. J. Neuroradiol..

[19] Selhorst JB, Chen Y (2009). The optic nerve. Semin. Neurol..

[20] Horsfield MA (2010). Rapid semi-automatic segmentation of the spinal cord from magnetic resonance images: Application in multiple sclerosis. NeuroImage.

[21] Pravatà E (2020). Influence of CNS T2-focal lesions on cervical cord atrophy and disability in multiple sclerosis. Mult. Scler. J..

[22] Jeffery G (1995). The human optic nerve: Fascicular organisation and connective tissue types along the extra-fascicular matrix. Anat. Embryol..

[23] Maniscalco JE, Habal MB (1978). Microanatomy of the optic canal. J. Neurosurg..

[24] Molloi S, Zhou Y, Kassab GS (2004). Regional volumetric coronary blood flow measurement by digital angiography: In vivo validation. Acad. Radiol..

[25] Sheth S, Branstetter BFT, Escott EJ (2009). Appearance of normal cranial nerves on steady-state free precession MR images. Radiographics.

[26] Jonas JB, Schmidt AM, Müller-Bergh JA, Naumann GO (1995). Optic nerve fiber count and diameter of the retrobulbar optic nerve in normal and glaucomatous eyes. Graefes Arch. Clin. Exp. Ophthalmol..

[27] Karim S, Clark RA, Poukens V, Demer JL (2004). Demonstration of systematic variation in human intraorbital optic nerve size by quantitative magnetic resonance imaging and histology. Invest. Ophthalmol. Vis. Sci..

[28] Jain NS (2015). Visualization of nerve fiber orientations in the human optic chiasm using photomicrographic image analysis. Invest. Ophthalmol. Vis. Sci..

[29] Bäuerle J (2013). Reproducibility and accuracy of optic nerve sheath diameter assessment using ultrasound compared to magnetic resonance imaging. BMC Neurol..

[30] Kim DH, Jun JS, Kim R (2018). Measurement of the optic nerve sheath diameter with magnetic resonance imaging and its association with eyeball diameter in healthy adults. J. Clin. Neurol..

[31] Geeraerts T (2008). Use of T2-weighted magnetic resonance imaging of the optic nerve sheath to detect raised intracranial pressure. Crit. Care.

[32] Kashiwagi K, Okubo T, Tsukahara S (2004). Association of magnetic resonance imaging of anterior optic pathway with glaucomatous visual field damage and optic disc cupping. J. Glaucoma.

[33] Liu C (2021). Optic nerve sheath diameter measured using magnetic resonance imaging and factors that influence results in healthy Chinese adults: A cross-sectional study. Chin. Med. J..

[34] Votruba M (2000). MRI of the intraorbital optic nerve in patients with autosomal dominant optic atrophy. Neuroradiology.

[35] Seitz J (2002). Magnetic resonance imaging in patients diagnosed with papilledema: a comparison of 6 different high-resolution T1- and T2(*)-weighted 3-dimensional and 2-dimensional sequences. J. Neuroimaging.

[36] Weigel M, Lagrèze WA, Lazzaro A, Hennig J, Bley TA (2006). Fast and quantitative high-resolution magnetic resonance imaging of the optic nerve at 3.0 tesla. Invest. Radiol..

[37] Zhang YQ (2012). Anterior visual pathway assessment by magnetic resonance imaging in normal-pressure glaucoma. Acta Ophthalmol..

[38] Yiannakas MC (2010). A method for measuring the cross sectional area of the anterior portion of the optic nerve in vivo using a fast 3D MRI sequence. J. Magn. Reson. Imaging.

[39] Ramli NM (2014). Novel use of 3T MRI in assessment of optic nerve volume in glaucoma. Graefes Arch. Clin. Exp. Ophthalmol..

