# Supplementary material for: Biometry extraction and probabilistic anatomical atlas of the anterior Visual Pathway using dedicated high-resolution 3-D MRI
Source: Sci Rep. 2024 Jan 3;14:453. doi: 10.1038/s41598-023-50980-x (PMC10764933; doi:10.1038/s41598-023-50980-x)

Biometry extraction and probabilistic anatomical atlas of the anterior Visual Pathway using dedicated high-resolution 3-D MRI.

Authors:

Emanuele Pravatà[1,8*†], Andrea Diociasi[2†], Riccardo Navarra[3], Luca Carmisciano[2], Maria Pia Sormani[2], Luca Roccatagliata[2], Andrea Chincarini[4], Alessandra Ossola[6], Andrea Cardia[6], Alessandro Cianfoni[1,8], Alain Kaelin-Lang[7,8], Claudio Gobbi[7,8] and Chiara Zecca[7,8]

Supplementary information

Supplementary figure S1. Dice similarity index (DSI) scores density distribution results from the leave-one-out spatial similarity validation. The median score was 0.85 (continuous line) with 95% of comparisons scoring > 0.77 (dashed line).

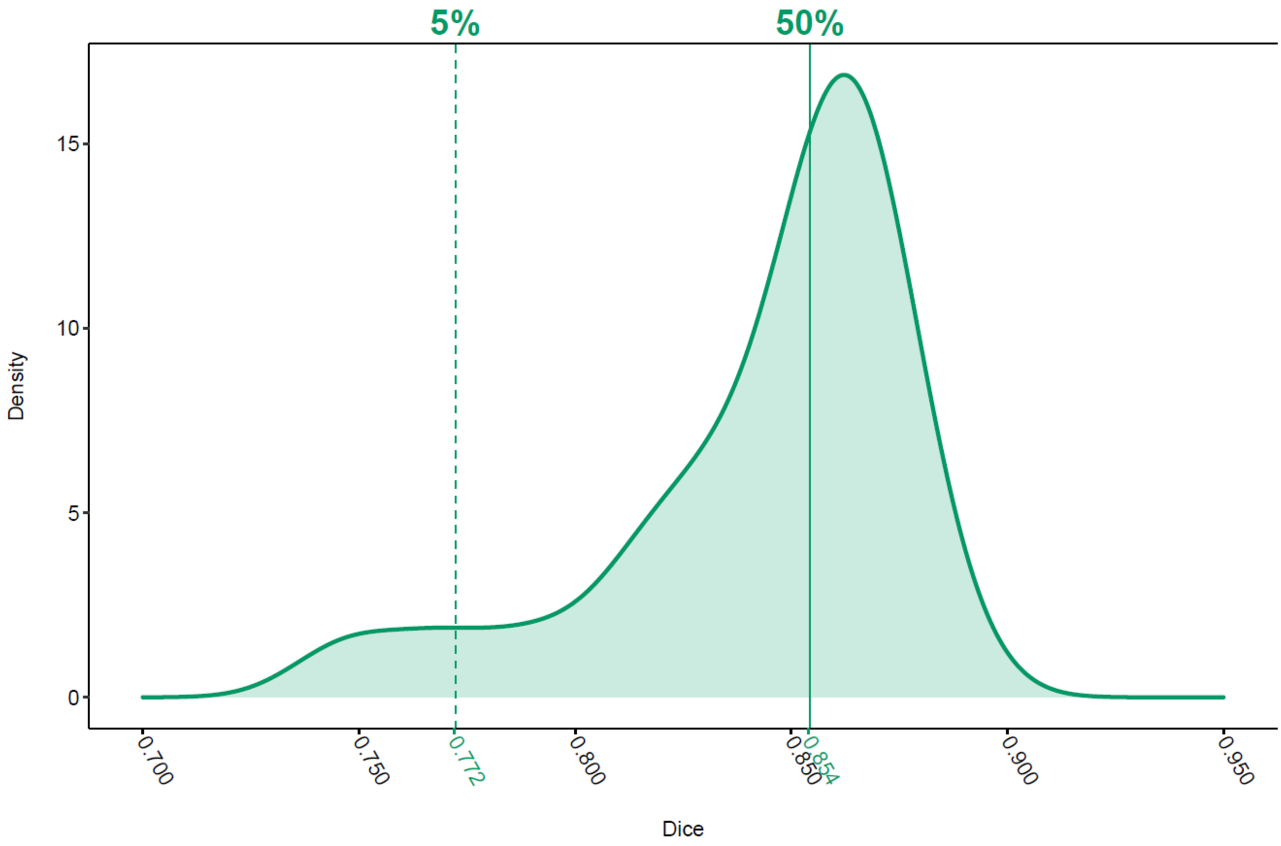

Supplement: Supplementary file 1 — Supplementary Information. [file 41598_2023_50980_MOESM1_ESM.pdf]
